# Neonatal Outcomes and Long-Term Follow-Up of Children Born from Frozen Embryo, a Narrative Review of Latest Research Findings

**DOI:** 10.3390/medicina58091218

**Published:** 2022-09-04

**Authors:** Giuseppe Gullo, Marco Scaglione, Gaspare Cucinella, Vito Chiantera, Antonino Perino, Maria Elisabetta Greco, Antonio Simone Laganà, Enrico Marinelli, Giuseppe Basile, Simona Zaami

**Affiliations:** 1 IVF Unit, Department of Obstetrics and Gynecology, Villa Sofia Cervello Hospital, University of Palermo, 90146 Palermo, Italy; 2 Department of Neuroscience, Rehabilitation, Ophthalmology, Genetics and Maternal-Child Sciences, University of Genoa, 16132 Genoa, Italy; 3 Unit of Gynecologic Oncology, ARNAS “Civico-Di Cristina-Benfratelli”, Department of Health Promotion, Mother and Child Care, Internal Medicine and Medical Specialties (PROMISE), University of Palermo, 90127 Palermo, Italy; 4 Department of Medico-Surgical Sciences and Biotechnologies, “Sapienza” University of Rome, 04100 Rome, Italy; 5 IRCCS Orthopedic Institute Galeazzi, 20161 Milan, Italy; 6 Department of Anatomical, Histological, Forensic and Orthopedic Sciences, “Sapienza” University of Rome, 00161 Rome, Italy

**Keywords:** fresh embryo transfer, frozen embryo transfer, cryopreservation, vitrification, neonatal outcomes

## Abstract

In recent years, the growing use of ART (assisted reproductive techniques) has led to a progressive improvement of protocols; embryo freezing is certainly one of the most important innovations. This technique is selectively offered as a tailored approach to reduce the incidence of multiple pregnancies and, most importantly, to lower the risk of developing ovarian hyperstimulation syndrome when used in conjunction with an ovulation-triggering GnRH antagonist. The increase in transfer cycles with frozen embryos made it possible to study the effects of the technique in children thus conceived. Particularly noteworthy is the increase in macrosomal and LGA (large for gestational age) newborns, in addition to a decrease in SGA (small for gestational age) and LBW (low birth weight) newborns. The authors aimed to outline a broad-ranging narrative review by summarizing and elaborating on the most important evidence regarding the neonatal outcome of children born from frozen embryos and provide information on the medium and long-term follow- up of these children. However, given the relatively recent large-scale implementation of such techniques, further studies are needed to provide more conclusive evidence on outcomes and implications.

## 1. Introduction

Gynecological diseases can lead to severe consequences for the quality of life of those affected [1]. Among these, infertility plays a central role. This is defined as the inability to conceive after at least one year of unprotected intercourse, and it has reached a global prevalence of 15% of couples at reproductive age [2]. The prevalence in the 1990–2017 period grew both for male (by 8.224% from 710.19 per 100,000 to 768.59 per 100,000, i.e., a 0.291% increase on a yearly basis) and female infertility (by 14.962% from 1366.85 per 100, to 1571.35 per 100 in 2017, a 0.370% increase every year) [3]. However, the increase in the prevalence of infertility and subfertility has led to an increasing demand for the use of assisted reproduction techniques. In particular, with the progress of conservation techniques in recent years, frozen embryo transfer has spread rapidly, giving rise to medical as well as ethical debates [4,5,6,7,8]. This technique, especially freezing by vitrification, lowers the likelihood of multiple pregnancies as well as the risk of incurring ovarian hyperstimulation syndrome. No significant differences have been found compared to transfer from fresh embryo in terms of pregnancy rate per cycle (63.1% vs. 60.9%) and the clinical pregnancy rate per cycle (55.4% vs. 58.7%) [9,10]. At the same time, as the technique has spread, several authors have investigated the possible issues related to it, such as the greater risk of hypertension syndrome and the increase in macrosomal and Large for Gestational age (LGA) newborns. The authors aimed to provide a comprehensive picture of the latest evidence regarding the neonatal outcome of babies born from frozen embryos. In addition, the authors have seen fit to try and shed a light on areas where current research data are still inconclusive. The further elaboration of still-indecisive findings (e.g., whether frozen cycles may result in higher live birth rates among patients with ovulatory disorder, or whether fresh cycles may be more designated for younger patients aged ≤ 30) would go a long way towards a higher degree of objectivity in the definition of evidence-based risk factors, which should be reflected in widely acknowledged guidelines and recommendations. Relying on broadly recognized criteria will in fact benefit clinical practice, patient care, and even shield doctors from malpractice claims, which are partly fueled by uncertainty and have increasingly affected healthcare professionals and OB/GYN operators in particular. Part of the review article will focus on the long-term outcome, particularly on the growth and development of children born from this technique. The search conducted by the authors ultimately identifies a total of 32 articles published in the 1987–2021 period, namely: 11 reviews/meta-analyses, 7 cohort studies, 12 retrospective cohort studies, and 2 randomized controlled trials. Most of the papers were published in 2020 (six), three in 2021, five in 2019, and five in 2018. The remaining ones were published in 2017, 2016, 2014, (two for each year, respectively), three in 2013, and only one each for years 2012, 2009, 2000, 1998, 1997, respectively. The selection criteria included papers in which the authors focused on the comparison of frozen embryo transfer births with fresh embryo births in terms of neonatal outcome and/or long-term development. The databases drawn upon by the authors were PubMed/Medline, Cochrane Database of Systematic Reviews, and Scopus and EMBASE; all were searched up to 2 February 2022 via the following search phrases: “Fresh embryo transfer”, “Frozen embryo transfer”, “adverse neonatal outcomes”, “infant birth weight”, “Assisted reproductive technology”, “In Vitro Fertilization (IVF)”, “ovarian stimulation”, “live birth”, ”ovarian hyperstimulation syndrome (OHSS)”, ”obstetric outcomes”. All studies that covered fresh vs. frozen embryo transfer from different perspectives than the ones specified above were excluded. The most significant findings from the various sources were schematized and points of agreement and divergence were discussed. Despite the degree of variability, inconsistency, and heterogeneity of findings between older studies and recent ones, the authors have set out to piece together a historical overview meant to reflect how research has progressed and evolved over the years as cryopreservation techniques have improved, thus offering new opportunities for tailored and targeted approaches. As an elaboration on the theme of cryopreservation, opinions of the authors on different neonatal outcomes between different freezing methods have been reported. Furthermore, six papers focusing on chromosomal abnormalities found in embryos obtained from frozen oocytes have also been included.

## 2. Neonatal Outcomes

**Figure 1:**
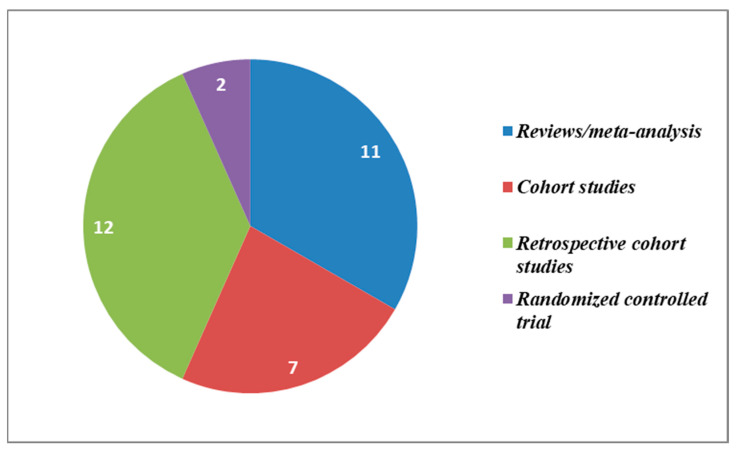
Studies distribution according to type of publication.

The following figure (Figure 1) sums up the research studies accounted for in this review according to the type of publication.

Table 1 summarizes the evidence reported by the various sources herein analyzed [11,12,13,14,15,16,17,18,19,20,21,22,23,24,25,26,27,28,29,30,31,32,33,34,35,36,37,38,39,40,41,42]. In particular, the aspects characterizing children born from frozen embryos compared to children born from fresh embryos have been highlighted. In the table, the section on perinatal outcomes and the section concerning congenital malformations in newborns and/or long-term outcomes of the children have been divided for the sake of clarity.

The topic of neonatal outcome is apparently much more broadly covered than congenital malformations and long-term outcome. In particular, 21 out of the 32 studies have focused exclusively on neonatal outcome, three exclusively on malformations and long-term outcome, and six on both aspects. Wennerholm et al. [41], in 1998, found similar gestational age at delivery, birthweight, the incidence of malformations, and the perinatal mortality between the frozen embryo transfer (FET) group and the fresh embryo transfer (ET) group both for singletons and twins, examining the medical records of 270 infants. The number of infants with Apgar score > 7 (calculated at the fifth minute) was similar, as was the percentage of intensive care admissions. Such data apparently point to a similar risk of adverse perinatal events between the two groups. In the following years, the aspect of neonatal outcome has been widely discussed by the authors and, as shown in Table 1, there is a common agreement in attributing a higher birth weight to newborns born from frozen embryos. The consequence is an increased number of macrosomic (>4500 g) and LGA newborns, in addition to a reduced number of LBW and SGA newborns [14,15,16]. Another aspect worth noting is the higher number of post-term infants accompanied by a reduced number of preterm infants. Certainly, the reduction of SGA and preterm infants solves one of the “side effects” associated with ART, though the increase in macrosomic/LGA infants entails different issues such as higher risk of stillbirth, fetal hypoxia, perineal lacerations, shoulder dystocia, cesarean section, postpartum hemorrhage, and neonatal metabolic disturbances at birth. For Belva et al. [32] and Liu et al. [36], the incidence of preterm infants in the two groups is comparable. In a 2016 cohort study, Belva et al. [32] reported that when considering twins instead of singletons, neonatal outcomes between the two groups are ultimately comparable. Ainsworth et al. [22] also reported a higher birth length and larger head circumference in children born from frozen embryos. An interesting retrospective cohort study published by Terho et al. [11] in 2021 differentiated neonatal outcomes based on sex and gestational age. Mean birth weights were significantly higher in the FET group compared to the fresh ET group starting from gestational week (GW) 33 for boys and from GW 34 for girls. In boys, there was a greater number of LGA births between GW 36 and 42, compared to those born from fresh ET. For girls, the same difference was found between GW 37 and 42. The proportion of SGA was significantly lower among boys born after FETs compared to fresh ETs between GW 36 and 42. For girls born after FET, the same difference was seen at GW 38 compared to those born after fresh ET. The percentage of LGA also was found to be significantly higher for boys born after FET between GW 38 and 41 and for girls born after FET between GW 37 and 40, if compared to boys and girls naturally conceived. For Maheshwari et al. [25], Belva et al. [32], Liu et al. [36], and Wennerholm et al. (in the 1997 paper) [42], the rate of perinatal death was also similar between the frozen embryo transfer (ET) group and the fresh ET group. This finding is very important if we consider the increased number of macrosomic infants in the frozen embryo group. Only one paper reported an increased rate of perinatal deaths [35] and only one reported a lower rate [27]. With regard to pregnancy rate and live birth rate, according to Acet et al. [13] and Orvieto et al. [16], the frozen ET groups seemed to have higher scores than fresh ET groups. However, in two cohort studies published in 2012 and 2013, Check et al. [37,38] found a higher rate of pregnancies successfully brought to term in fresh ETs, although the use of FET has been linked to a lower risk of ovarian hyperstimulation syndrome. In a review published in 2017, Wong et al. [27] found similar cumulative live birth rates. Furthermore, LGA newborns are common for patients with insulin resistance and polycystic ovarian syndrome (PCOS) that could be preemptively treated with inositol supplementation [43,44], as could patients with GD (gestational diabetes) to improve birth outcomes [45], as well as young women who decide to preserve their oocytes and have a FET close to menopausal age [46].

Table 2 summarizes the comparison between the two techniques with regard to neonatal outcomes.

### 2.1. Differences in Neonatal Outcomes between Freezing Methods

The recent improvements in freezing techniques has led to a gradual abandonment of the slow-freeze technique with cleavage stage embryos in favor of vitrification at the blastocyst stage. Studies comparing the outcomes of the two techniques are still few and with conflicting conclusions. Ginström Ernstad et al. [21] conducted a cohort study published in 2019 in which they found comparable neonatal outcomes in children born from the two different techniques. However, Liu et al. [36], comparing techniques, found a median birthweight from vitrified embryos (3455.3 g) higher than those from slow freezing (3352.3 g) and fresh (3355.8 g) transfers. The rate of perinatal mortality is instead reported as comparable between the three groups.

Moreover, in a cohort study published in 2014, Li et al. [34] suggested that the freezing method can influence neonatal outcome; in particular, they found an higher clinical pregnancy rate in vitrified blastocyst transfer cycles than in slow frozen blastocyst transfer cycles.

In addition, Alviggi et al. [47] suggested that the freezing method and the time of transfer may influence pregnancy outcomes in terms of preterm birth, very preterm birth, LGA, SGA, and perinatal mortality.

### 2.2. The Role of Confounding Factors

Although most authors agree on the data regarding birth weight, doubts do arise in some studies. According to Ainsworth et al. [19], in fact, there is no difference in birth weight when adjusting for gestational age, sex, and maternal factors. However, Vidal et al. [30], in a 2017 cohort study, argue that adjusted regression model birthweight is significantly higher in the fresh ET group than the frozen one. In a retrospective cohort study published in 2019, Maris et al. [23] also found a higher birthweight after a multivariate analysis adjusted according to confounding factors such as gestational age, maternal age, maternal body mass index (BMI), tobacco exposure, the number of embryos transferred, and birth order. In a 2014 cohort study, Pinborg et al. [33] argued that the increased risk of LGA newborns could not be related exclusively to intrinsic maternal factors, but must necessarily be related, at least for the most part, to the freezing procedure. According to Pirtea et al. [14], the difference found in neonatal outcomes derives from issues regarding the depth of placentation, possibly being too shallow in the fresh ET group. For Berntsen et al. [26], further studies are needed to define what changes, probably epigenetic, may stem from frozen embryo transfers.

### 2.3. Congenital Malformations and Long-Term Outcome in Children

In a 1997 study by Wennerholm et al., 255 children from cryopreserved embryos were matched (regard to maternal age, date of delivery, and parity, single or twin pregnancy), with 255 children born after IVF with fresh embryos, and 252 children from spontaneous pregnancies [42]. Growth features were similar for both singletons and twins in the three groups. There were six (2.4%) major malformations in the cryopreserved group, nine in standard IVF group (3.5%), and eight (3.2%) in naturally conceived group.

The prevalence of chronic diseases during infancy and early childhood did not differ between the three groups (18.0%, 15.3%, and 16.7% in the cryopreserved group, standard IVF, and spontaneous groups, respectively). In that paper, occurrences of minor behavioral disturbances, learning difficulties, and attention and perception deficits were not reported because of too young an age of the children involved. In the following years, as mentioned above, few studies focused on the long-term health outcomes not exclusively neonatal of children born from frozen embryos. In an RCT published in 2020, Vuong et al. [15] performed follow-ups of children in the study group (consisting of 391 pairs) until an age of 37 months. Developmental screening was performed using the well-known ASQ-3 questionnaire that covers 5 domains: communication, gross motor, fine motor, problem solving, and personal social behavior. The study reported relevant findings: problem solving scores were found to be higher in the frozen ET group than in the fresh ET group, but not when singletons and twins were analyzed separately. Other data in favor of the frozen ET group concerned the fine motor skills in the overall analysis (*p* = 0.056 vs. fresh ET) and twins (*p* = 0.06 vs. fresh ET) but not in singletons. There were no significant differences in the prevalence of abnormal ASQ-3 scores found among the study groups. This finding is important and indicates that there is no difference in the incidence of neurodevelopmental abnormalities, although for some developmental domains, the scores of children born from frozen embryos are even better. The few data available, however, do not allow for a determination as to whether any difference exists when considering singletons separately from twins.

The same aspect has been evaluated by Djuwantono et al. [18] in a 2020 review; in particular, these authors do not report a higher rate of neurodevelopmental abnormalities in children born after frozen embryo transfers. The already-mentioned prospective study by Belva et al. [32] collected data from 960 cycles after frozen embryo transfers and 1644 cycles after fresh embryo transfers, performed between 2008 and 2013. Follow-up was performed in the 3 months after birth with a close focus on congenital malformations. Children’s pediatricians were blinded to the transfer method. Data were adjusted for treatment variables and maternal characteristics. The mothers of the children belonging to the frozen ET group tended to be older and more prone to pregnancy-related hypertension, a finding already known in the literature. As for the frequency of major congenital malformations in live births (i.e., malformations that have both a morphological and functional impact), it was found to be comparable between the vitrified group and the fresh group, both among singletons and twins. Even considering major and minor malformations together, the study groups had similar rates. Zhang et al. [28], in a 2018 retrospective cohort study, and Maheshwari et al. [25] in a 2018 review also reported similar congenital malformation rates between frozen ET groups and fresh ET groups. Ainsworth et al. [22] focused on child growth by including 136 women in the study, 87 of whom underwent a fresh embryo transfer and 49 a frozen embryo transfer. Age- and sex-specific weight and body mass index results, considering percentiles, were comparable between the study groups.

Only one retrospective cohort study published in 2019 reported a comparison regarding other health outcomes. Significantly, such a study found that babies born from frozen embryos had greater odds of infectious disease (AOR = 1.46), respiratory conditions (AOR = 1.23), and neurological (AOR = 1.32) conditions. No statistically significant differences were found for birth defects, cardiovascular, hematologic, and gastrointestinal/feeding conditions. [24]

In light of the limitations due to the dearth of currently available research data, Table 3 summarizes the long-term follow-up of children born from frozen embryos.

## 3. Concluding Remarks

This narrative review has been conceived to elaborate on the latest research data regarding the neonatal and long-term developmental outcomes of children born from frozen embryos, which were collected and weighed against the outcomes of children born from fresh embryo transfers.

The increased presence of macrosomic and LGA newborns certainly reduces the proportion of SGA newborns, but it requires greater attention during childbirth maneuvers and in the monitoring of neonatal problems. LGA newborns have rarely been found in cases of perinatal infections [48].

The increase in pregnancy rate and live birth rate are certainly reassuring aspects as to the use of frozen embryo transfer, as is the lower risk of ovarian hyperstimulation syndrome and multiple pregnancies. Such favorable outcomes may be partly due to the fact that frozen embryo transfers make it possible to wait until the ovary has recovered from the ovarian stimulation and the exposed endometrial lining has shed, thus enabling a “fresh start” for both. In fact, higher OHSS risks and pregnancy loss have been linked to higher estradiol levels following IVF. It is worth noting in that regard that estradiol levels in fresh cycles are considerably higher than in frozen cycles [31,49]. Since frozen embryos would be implanted long after ovulation induction, the mother’s body would have had the chance to get back to normal conditions from the hormonal standpoint. Such newfound normalcy is thought to better reproduce the natural conception path associated with a higher likelihood of success. Moreover, better planning made possible by the use of frozen embryos enables the patient to have the embryo transferred at the ideal time. At any rate, the differences found between the two groups are unlikely to be related to mother-dependent factors. Of the studies herein examined, only one accounts for length and head circumference in addition to birth weight, showing the two parameters to be greater in the FET children. With regard to perinatal morbidity and mortality, the risk appears to be similar between the two groups. Only one study reported a higher perinatal death rate in the FET group and only one reported a lower rate in the FET group compared to the fresh ET group.

As for the rate of congenital malformations, in the five studies that dealt with this topic, all the authors reported comparable malformation rates between the two groups. This finding appears reassuring with regard to the use of freezing techniques.

Another important finding concerns the incidence of neurodevelopmental abnormalities, with apparently no difference between the two groups. This finding, in addition to the most recent scientific evidence regarding the safety of ART on the neuro-psychomotor outcome of newborns, is important and reassuring for all couples with infertility and sub-fertility problems. In a single study, randomized, controlled evidence has emerged reflecting improved cognitive performance in children born from frozen embryos, at least in some specific domains. Although significant, such data must be contextualized because they were not found by separate analyses of twins and singletons. With regard to the growth of children born from frozen embryos, two studies investigating the issue found weight, BMI (normalized on age and sex), and other growth parameters to be similar in the two study groups. This finding is meaningful in that it suggests that a higher incidence of macrosomic newborns in frozen embryo babies does not necessarily result in worse long-term health outcomes in such children.

Only one study of the aforementioned reported a greater risk in newborns of incurring infectious diseases and respiratory or neurological abnormalities. The data reproducibility needs further research. In conclusion, research data on neonatal outcomes from frozen embryos are varied and evidence-based. On the contrary, the lack of data on the long-term follow-up of children requires further in-depth studies. With regard to the study of neurodevelopmental alterations, prospective studies with an adequate number of patients should be prioritized (given the rarity of some disorders), setting up a clinical monitoring framework that should cover not only the first years of life, but also the school age in which mild neurodevelopmental disorders (non-pervasive) may appear that are not easily recognizable in early childhood and preschool age. With regard to congenital malformations, future studies should be focused on individual organs that are possibly affected, e.g., rates of congenital heart disease, renal/urinary malformation, or malformations of the digestive system. Reliance on more objective, factual, and evidence-based data would also go a long way towards enabling doctors to be safe from legal malpractice claims in case of adverse outcomes. Such lawsuits are in fact particularly common and often severely burdensome for OB/GYN professionals. The delineation of broadly acknowledged guidelines and best practices is in fact instrumental in providing a degree of objectivity through which healthcare professionals can abide by and document their compliance with recognized criteria if called to answer for adverse outcomes.

## Figures and Tables

**Table 1:** Studies description regarding to the main findings on neonatal outcomes and child growth and/or development after transferring frozen versus fresh embryos.

Frozen and Vitrified Embryos vs. Fresh Embryos				
Authors (et al.)	Type of Study	Year	Neonatal Outcomes	Child Growth and/or Development
Terho et al. [11]	Retrospective cohort	2021	More LGA newborns and higher birth weight both in boys and girls	Not evaluated
Zaat et al. [12]	Review	2021	More LGA newborns and higher birth weight	Not evaluated
Acet et al. [13]	Retrospective cohort	2021	Higher pregnancy rate and live birth rate	Not evaluated
Pirtea et al. [14]	Review	2020	More LGA and fewer SGA newborns; less premature and more postdate births	Not evaluated
Vuong et al. [15]	RCT	2020	Not evaluated	Better ASQ-3 score and motor skills scores
Orvieto et al. [16]	Review	2020	Higher live birth rate; fewer premature and LBW babies; more LGA newborns	Not evaluated
Chen et al. [17]	Retrospective cohort	2020	Higher birthweight in live- born twins	Not evaluated
Djuwantono et al. [18]	Review	2020	Not evaluated	Not higher risk of neurodevelopmental disorders
Elias et al. [19]	Review and meta-analysis	2020	More LGA newborns; fewer SGA and LBW newborns	Not evaluated
Ginström Ernstad et al. [20]	Cohort	2019	More LGA and macrosomic newborns	Not evaluated
Ginström Ernstad et al. [21]	Retrospective cohort	2019	More LGA and macrosomic newborns; fewer premature and LBW newborns	Not evaluated
Ainsworth et al. [22]	Cohort	2019	Higher birth length, weight, and head circumference	No significant differences in age/sex specific weight and BMI
Maris et al. [23]	Retrospective cohort	2019	Higher birth weight (even after adjustment)	Not evaluated
Hwang et al. [24]	Retrospective cohort	2019	More LGA newborns and higher birth weight; fewer SGA newborns	Increased odds of infectious disease, respiratory, and neurologic abnormalities.
Maheshwari et al. [25]	Review	2018	Higher BW, fewer SGA and preterm babies; not differences in perinatal deaths	No significant differences in congenital malformations
Bernsten et al. [26]	Review	2018	More LGA and macrosomic newborns; fewer premature and LBW newborns	Not evaluated
Sha et al. [27]	Review	2018	Fewer LBW, SGA newborns and perinatal deaths	Not evaluated
Zhang et al. [28]	Retrospective cohort	2018	More LGA and macrosomic newborns; fewer premature and LBW newborns	No significant differences in congenital malformations
Wong et al. [29]	Review	2017	Similar cumulative live birth rates	Not evaluated
Vidal et al. [30]	Cohort	2017	Fewer premature and LBW newborns	Not evaluated
Chen et al. [31]	RCT	2016	Higher live birth rate; no differences in neonatal complications	Not evaluated
Belva et al. [32]	Cohort	2016	Higher BW and fewer SGA newborns; no differences in preterm births and perinatal death rate; similar neonatal outcomes if considered twins	No significant differences in congenital malformations both in twins and singletons
Pinborg et al. [33]	Cohort	2014	More LGA newborns	Not evaluated
Li et al. [34]	Cohort	2014	Fewer preterm and LBW newborns	Not evaluated
Wennerhom et al. [35]	Retrospective cohort	2013	More LGA, macrosomic newborns, postdate births and perinatal deaths; fewer premature and LBW newborns	Not evaluated
Liu et al. [36]	Cohort	2013	Higher birthweight, fewer LBW newborns; no differences in preterm and perinatal death	Not evaluated
Check et al. [37]	Retrospective cohort	2013	Higher live-delivered pregnancy rate	Not evaluated
Check et al. [38]	Retrospective cohort	2012	Higher live-delivered pregnancy rate	Not evaluated
Wennerhom et al. [39]	Review	2009	Fewer preterm and LBW newborns	No significant differences in congenital malformations
Wennerhom [40]	Review	2000	Not significant differences in perinatal outcome	No significant differences in congenital malformations nor child development
Wennerhom et al. [41]	Retrospective cohort	1998	Not evaluated	No significant differences in growth and chronic diseases
Wennerhom et al. [42]	Retrospective cohort	1997	Similar perinatal risk	Not evaluated

ASQ-3: Ages & Stages Questionnaires ^®^, Third Edition. BMI: body mass index. LGA: large for gestational age; SGA: small for gestational age; LBW: low birth weight; RCT: randomized controlled trial. BW: birth weight. LBW: low birth weight.

**Table 2:** Comparison of neonatal outcomes between frozen embryo and fresh embryo methods according to the frequency.

Compared Neonatal Outcomes between Freezing Methods		
Frozen Embryo > Fresh Embryo	Frozen Embryo = Fresh Embryo	Frozen Embryo < Fresh Embryo
Live births	Perinatal Deaths	LBW newborns
Obtained pregnancies		SGA newborns
Macrosomic newborns		Premature Births
LGA newborns		
Postdate births		

LGA: large for gestational age; SGA: small for gestational age.

**Table 3:** Comparison of long-term health aspects in children between frozen embryo and fresh embryo methods.

Compared Long Term Follow-Up in Children between Freezing Methods		
Frozen Embryo > Fresh Embryo	Frozen Embryo = Fresh Embryo	Frozen Embryo < Fresh Embryo
Problem solving scores	Congenital malformations rate	
Fine motricity scores	ND prevalence	
	Age- and sex-specific BMI and weight	
	Growth and chronic diseases	

BMI: body max index; ND: neurodevelopmental disorders.

## Data Availability

The data presented in this case report are available on request from the corresponding author.
